# Carbon-Based Electrode Materials for Microsupercapacitors in Self-Powering Sensor Networks: Present and Future Development

**DOI:** 10.3390/s19194231

**Published:** 2019-09-29

**Authors:** A. D. Smith, Qi Li, Agin Vyas, Mohammad Mazharul Haque, Kejian Wang, Andres Velasco, Xiaoyan Zhang, Shameel Thurakkal, Arne Quellmalz, Frank Niklaus, Kristinn Gylfason, Per Lundgren, Peter Enoksson

**Affiliations:** 1 Micro and Nanosystems Group, Department of Microtechnology and Nanoscience, Chalmers University of Technology, 41296 Gothenburg, Sweden; qili@chalmers.se (Q.L.); agin@chalmers.se (A.V.); mhaque@chalmers.se (M.M.H.); kejian@student.chalmers.se (K.W.); andres.velasco.13@gmail.com (A.V.); per.lundgren@chalmers.se (P.L.); peter.enoksson@chalmers.se (P.E.); 2 Chemistry on 2D Materials Group, Department of Chemistry and Chemical Engineering, Chalmers University of Technology, 41296 Gothenburg, Sweden; xiaoyan.zhang@chalmers.se (X.Z.); shameel@chalmers.se (S.T.); 3 Department of Micro and Nanosystems, School of Electrical Engineering and Computer Science, KTH Royal Institute of Technology, SE-10044 Stockholm, Sweden; arneq@kth.se (A.Q.); frank.niklaus@ee.kth.se (F.N.); gylfason@kth.se (K.G.)

**Keywords:** microsupercapacitors, energy storage, self-powering systems, IoT, sensor networks

## Abstract

There is an urgent need to fulfill future energy demands for micro and nanoelectronics. This work outlines a number of important design features for carbon-based microsupercapacitors, which enhance both their performance and integration potential and are critical for complimentary metal oxide semiconductor (CMOS) compatibility. Based on these design features, we present CMOS-compatible, graphene-based microsupercapacitors that can be integrated at the back end of the line of the integrated circuit fabrication. Electrode materials and their interfaces play a crucial role for the device characteristics. As such, different carbon-based materials are discussed and the importance of careful design of current collector/electrode interfaces is emphasized. Electrode adhesion is an important factor to improve device performance and uniformity. Additionally, doping of the electrodes can greatly improve the energy density of the devices. As microsupercapacitors are engineered for targeted applications, device scaling is critically important, and we present the first steps toward general scaling trends. Last, we outline a potential future integration scheme for a complete microsystem on a chip, containing sensors, logic, power generation, power management, and power storage. Such a system would be self-powering.

## 1. Introduction

Electronic devices have been shrinking for decades in accordance with Moore’s law—providing additional processing power and storage capacity with each technological iteration [1]. However, recent trends in industry, as well as physical limits of silicon technology suggest that Moore’s law is nearing its end [2]. In consequence, manufacturers are searching for ever more inventive ways to continue technological progression, such as energy efficient architectures [3], three-dimensional (3D) stacking, and the addition of multiple cores, to speed up processing [2,4]. Simultaneously, as devices continue to have increased computational power, they experiencing a complimentary increase in functionality (diversification), which has come to be referred to as ‘more-than-Moore’ [2,5,6]. Diversification involves the incorporation of a wide variety of sensor components into one device—typically through a combination of system on chip (SoC) and system in package (SiP) solutions [6]. A consequence of additional functionality is an increasing power requirement. Additionally, most applications using more-than-Moore technologies, such as smart phones and wearable electronics, benefit greatly from having access to energy storage, which is quickly recharged with insignificant degradation over the device’s lifetime.

Batteries have been commonly incorporated for commodity storage applications [7,8,9]. However, although they have high energy density, batteries suffer from poor cyclability [10] and slow recharging rates. In contrast, a relatively novel device structure, the supercapacitor, offers excellent cyclability and promises charge/discharge rates in the order of seconds [11] rather than hours—making supercapacitors an attractive alternative to batteries for future more-than-Moore applications. Supercapacitors operate primarily by employing a double-layer capacitance, meaning that ions collect along the electrode surface and attract their charge pair from the electrolyte. Consequently, materials with a high surface area can achieve a correspondingly high energy density. As such, there has been significant research investigating both wearable and flexible electronic supercapacitor applications [12,13,14,15,16], as well as microsupercapacitors (MSCs) for more-than-Moore applications.

One particularly attractive application involves the integration of supercapacitors with energy harvesters. Such a device combination is potentially self-powering [17,18,19,20], as excess energy from the harvester is stored in the supercapacitor for use at times of intermittent energy generation from the harvester. A combined supercapacitor and harvester component can then be connected to sensor/digital logic/memory systems and power those systems indefinitely. 

Currently, supercapacitors have energy densities that are far lower than batteries. Typical commercial batteries have energy densities of 140 Wh/kg at the battery pack level [21], while commercial supercapacitors have reported energy densities that are as high as 30 Wh/kg [22]. Consequently, a substantial portion of the research into supercapacitors has been devoted to increasing their energy density. The best reported values for supercapacitors, that we are aware of, exceed 200 Wh/kg at the cell level [23,24], which is comparable to the commercially available battery technology. There are a number of ways to improve supercapacitor device performance which include asymmetric electrode designs, hybrid supercapacitor/battery designs [25,26,27,28,29], electrolyte optimization [22,30,31], and novel electrode materials [22,30,32,33,34,35,36,37,38] including chemical doping of electrodes [39]. Graphene-based materials have shown a great promise as electrode materials, due to their high electrical conductivity, large surface-to-volume ratio and excellent flexibility [36,40,41,42]. Therefore, a number of studies have been devoted to examining graphene-based materials as electrode materials for supercapacitor applications [43,44,45,46,47,48,49,50].

**Figure 1:**
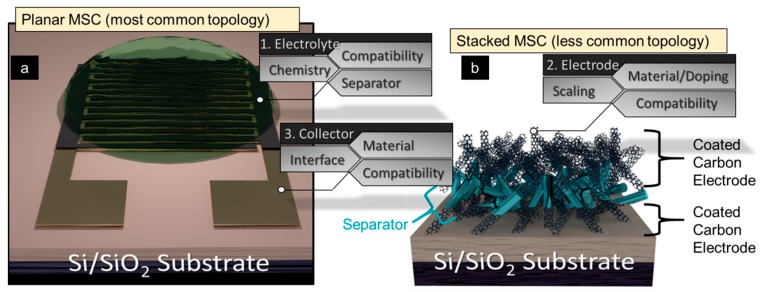
Schematic of a microsupercapacitors (MSCs)—planar (**a**) and stacked (**b**)—and its design parameters. There are 3 primary areas for performance improvement—electrolyte, electrode, and current collector. Each of these areas have 3 subset groups for improvement. This work aims to provide an overview of each area and subset group.

The present article continues investigations into the application of graphene-based materials for supercapacitor applications, including a focused review and a report of our own design solutions to key challenges. We outline recent developments while providing an overall context of MSC design parameters and our current attempts at performance improvement. Figure 1 outlines three main areas for improvement (electrode, electrolyte, and current collector) and the two primary MSC topologies (planar, Figure 1a, and stacked, Figure 1b). We present our current progress at improving these areas and provide additional paths for future investigation. Although there are a number of electrode materials available [32], the primarily examined materials here are reduced graphene oxide (rGO) and vertically grown graphene. The contact resistances between graphene and current collectors are also critically important, as it impacts the energy losses of each charge/discharge cycle of the MSC. Hence, this work provides an analysis of graphene/current collector contact resistances under the influence of moisture, as electrodes will likely be immersed in liquid electrolyte and be subjected to moisture during their lifetime. Additionally, the implementation of chemical doping into the electrode materials is likely to improve the energy density of the supercapacitor, without any critical penalty on power density and cyclability. Consequently, this work presents the potential options for doping electrodes with redox-active heteroatoms. Based on the device topology, the separator can play an important role, hence, its importance is discussed. Geometric scaling of components is also examined and it represents an important incremental step in effectively engineering the desired performance of the MSC.

## 2. Materials and Methods

### 2.1. Planar Microsupercapacitor Fabrication

**Figure 2:**
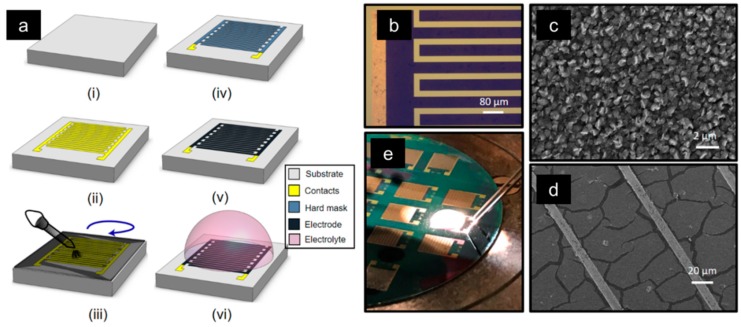
(**a**) Schematic CMOS compatible process for MSC fabrication. (**b**) Optical micrograph of the fabricated intedigitated reduced graphene oxide (rGO) electrodes on Au/Ti current collectors. (**c**) Graphical SEM image of vertical graphene on a 50 mm diameter Si/SiO_2_ wafer with Au/Ti collectors. (**d**) SEM image of rGO on Fe/Ti/Au current collectors. (**e**) Electrochemical test setup of fabricated rGO-MSCs with H_3_PO_4_/polyvinyl alcohol (PVA) electrolyte.

MSCs can be designed and fabricated in various ways, depending on their target application [35]. For example, flexible electronics requires foldable MSCs, which must demonstrate a stable performance under strain [51,52]. Several manufacturing approaches, such as spray coating [53,54], electrophoretic deposition [55,56], and laser patterning of thin-film electrodes [57,58,59], have been used to coat electrode materials onto a wide variety of target substrates (standard CMOS substrates such as SiO_2_ or flexible substrates such as polydimethylsiloxane (PDMS). The integration of supercapacitors with CMOS technology, requires coating and patterning methods compatible with CMOS circuits, including temperatures below 450 °C. In this work, we primarily focused on MSC fabrication using a planar design (although we briefly elaborated on stacked configurations in a later section) and a fully CMOS-compatible process (Figure 2a). The process begins with a Si/SiO_2_ wafer (Figure 2a(i)) which could in principle also be a wafer with prefabricated CMOS circuits. Current collectors are patterned on it, either through a bi-layer metal lift-off process or through a metal-etching post-blanket-wafer-evaporation (Figure 2a(ii)). Some current collector materials, such as Au or Pd, require a diffusion barrier layer between them and an insulating substrate, as some metals tend to migrate into the pin-holes in the insulating passivation layers—short circuiting the device.

Electrodes can either be deposited or grown on the substrate, depending on the choice of the material. Techniques such as doctor-blade coating and spin-coating are effective in covering the entire wafer surface for materials such as carbon nanotubes (CNTs), reduced graphene oxides (rGOs), carbon nanofibers (CNFs), and activated carbons (ACs). Growth of CNTs, CNFs, and vertical graphene (VG) is also possible on the wafer (Figure 2a(iii)). Each method has its own advantages and disadvantages. For example, spin-coating is challenging, both for achieving a strong adhesion of the electrode to the substrate and to obtain a uniform coating. Likewise, direct growth on pre-patterned substrates is typically challenging, due to the uneven temperature distribution across the surface. In this work, we focus on spin-coated rGO and direct growth of VG. After the electrode deposition, a metal, such as Al, is evaporated onto the electrode to act as a hard mask (Figure 2a(iv)). The exposed areas of the electrode are then etched using an O_2_-reactive ion-etching (Figure 2a(v)), followed by a removal of the hard mask (Figure 2a(vi)). Lastly, an electrolyte is drop-cast onto the surface of the MSC. 

### 2.2. Electrode Materials

**Figure 3:**
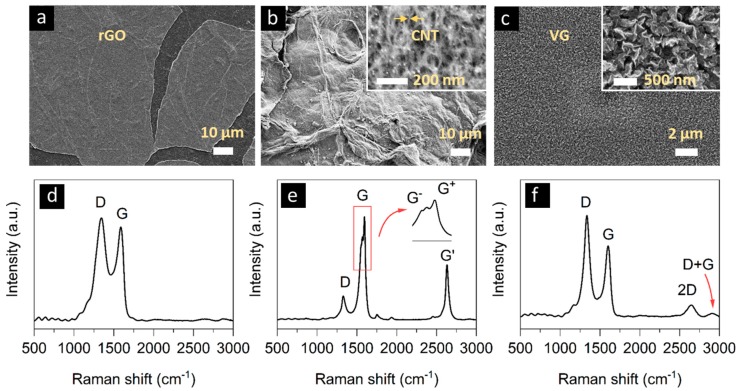
SEM images of (**a**) rGO, (**b**) carbon nanotubes (CNT), and (**c**) a vertical graphene (VG) layer as electrode materials on an SiO_2_ surface. Insets show higher magnification of the respective sample. Raman spectroscopy images of each material are shown in (**d**), (**e**), and (**f**), respectively.

As previously mentioned, to be compatible with the top-down approach for microsupercapacitor manufacturing, the active electrode, collector, and separator materials must be deposited onto the surface of wafers. The deposition of the electrode material can be achieved through two different strategies, i.e., spin-coating and a direct growth of materials [17,36,44]. For the spin coating route, stable inks containing supercapacitor active materials must be prepared. To this end, we have successfully demonstrated graphene oxide (GO) and carbon nanotubes (CNTs). For the direct growth, we have uniformly grown vertical graphene (VG) on wafer surfaces. Representative SEM images of the respective investigated electrode approaches are shown in Figure 3. 

Additionally, Raman spectra of rGO, CNT, and VG are shown in Figure 3d, e, and f, respectively. Two fundamental vibrations can be found for rGO, as shown in Figure 3d. The D band originated from a breathing mode of j-point photons of A1g symmetry, and the G band is from first-order scattering of E2g phonons by sp^2^ carbon [60], as well as C–C stretching [61]. The intensity ratio of G to D bands (I_G_/I_D_) is correlated with the amount of defects in graphitic materials [62]. The ratio is calculated to be 1.10 for rGO and is comparable to other reported rGO materials [63]. Raman spectrum of CNTs is shown in Figure 3e. D, G, and G’ bands can be recognized at about 1327, 1594, 2631 cm^−1^, respectively. Noticeably, the G band is split into G+ and G– bands, attributed to a slight carbon sheet curvature and is considered to be a signature of CNTs [48]. The ID/IG ratios is 0.23, indicating a relatively low defect concentration for the CNTs. Figure 3f displays typical features of VG—besides the D and G bands, a secondary D (2D) band is observed at about 2648 cm^−1^, and a G + D band [64] at around 2913 cm^−1^. The ID/IG ratio is about 1.43 for the VG used in this work, comparable to previous reports [65].

#### 2.2.1. Graphene Oxide (GO)

Highly concentrated single layer GO solution was purchased from Graphene Supermarket. The commercial GO solution is prepared through a modified Hummers method by reacting graphite with a mixture of potassium permanganate (KMnO_4_), concentrated sulfuric acid (H_2_SO_4_), and sodium nitrate (NaNO_3_). The oxygen-containing surface functional groups have a high affinity to water molecules, therefore, GO is hydrophilic and ‘dispersible’ in water. The flake size of GO ranges from 0.5 to 5 μm, with a single layer GO content of over 80%. The purchased water-based ink has a concentration of 6.2 g/L and was later diluted to 3 g/L, by adding deionized water.

Due to the insulating nature of GO caused by the disrupted sp^2^ bonding network, a reduction step is required to recover most of the properties of graphene. GO reduction can be done through chemical, thermal, or electrochemical means. In the present study, the GO was thermally or chemically reduced. Besides reduction of oxygen-containing groups, exfoliation of stacked GO occurs at a high temperature, due to gas (e.g., CO_2_) production—improving the surface area [66]. 

#### 2.2.2. Carbon Nanotubes (CNTs)

CNTs have a narrow distribution of size (diameter) in the nanometer range, a highly accessible surface area, high conductivity, and stability. These features are promising for supercapacitor applications. However, CNTs tend to form large agglomerates, making the processing and stabilization of CNT-containing inks for spin-coating challenging. To counter this problem, external energy needs to be supplied to overcome the internal forces holding the aggregates together. Moreover, after the CNTs detach from the aggregates, there is a possibility of re-agglomeration. Therefore, a suitable type of surfactant is needed to stabilize them in water-based solutions.

In the present study, the CNT dispersions were prepared according to a previous work [67]. In general, 30 mg of CNTs was dispersed in 15 mL of water. The dispersion was prepared by first stirring the CNTs with a 7 mL water at 90 °C in a water bath, for 1 h, followed by 20 min sonication to increase the energy for de-agglomeration. Afterwards, cetyltrimethylammonium bromide (CTAB), which was used as a surfactant to prepare a stable dispersion, was added in an 8 mL solution form. By keeping the mixture at 90 °C for another 1 h under constant stirring and 20 min of sonication, the dispersion was centrifuged to remove the undispersed CNTs. 

#### 2.2.3. Vertical Graphene (VG)

Horizontal graphene has poor out-of-plane conductivity and a limited accessible area for capacitive energy storage, thereby, limiting its performance in microsupercapacitors. In contrast, vertical graphene has a high out-of-plane electrical conductivity, large surface-to-volume ratio, and an open network structure with graphene-like flakes oriented perpendicularly to the substrate. The vertical arrangement was beneficial for capacitive energy storage, due to a high-ion diffusivity and ion accessibility. 

In this work, we implemented a plasma-enhanced chemical vapor deposition (PECVD) process to grow VG. Hydrogen, argon, and acetylene gases were used as a reducing agent, carrier gas, and carbon source, respectively, for the growth. The wafer substrate was heated up to 775 °C under argon and hydrogen, and then acetylene was introduced to grow the VG for 10 min under an 80 W direct current plasma.

## 3. Results

### 3.1. Electrode Adhesion

Spin-coated electrode materials can suffer from issues related to poor adhesion to the wafer substrate and current collectors. These issues are two-fold. First, the material deposited through spin-coating is non-uniform. The thickness of the fabricated electrodes varies substantially. This leads to an uneven device performance across devices on the wafer. Second, the fabrication of MSCs involves submersion of the wafer into various developers. If the electrodes do not adequately adhere to the surface, the process leads to a loss of electrode material. This eventually leads to a poor device manufacturing yield. There are two main causes for these issues. The substrates are typically Si wafers covered with an insulating thermally grown thin-film of SiO_2_. On top of this, metal current collectors are deposited and patterned. The current collector and SiO_2_ surfaces typically do not have the surface roughness necessary to ensure a reliable adhesion of the electrode flakes onto the surfaces.

**Figure 4:**
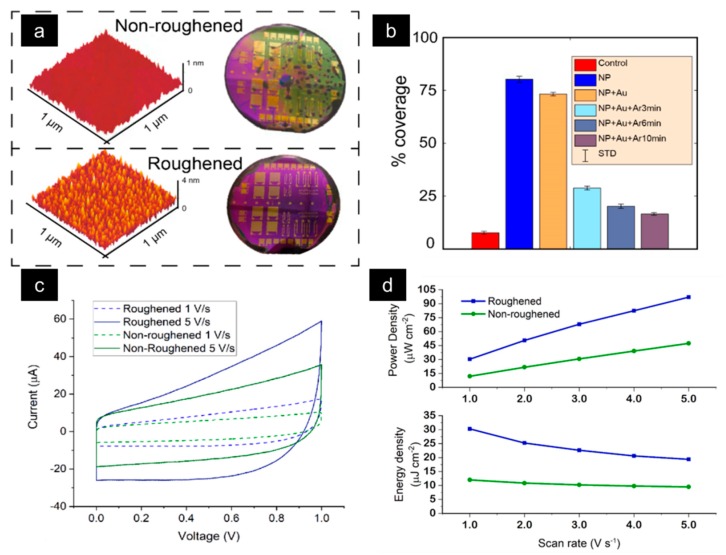
(**a**) AFM comparison of roughened and non-roughened sample surfaces along with optical microscopy images showing the coverage of the electrode spin-coating. (**b**) rGO spin-coated chips under different design variations (percentages of surface coverage evaluated from the optical microscopy images). Note that all roughened samples outperform the non-roughened. (**c**) Cyclic voltammograms, and (**d**) energy and power densities of the fabricated roughened and non-roughened MSCs with 1-Ethyl-3-methylimidazolium Bis(trifluoromethanesulfonyl)imide (EMIM-TFSI) electrolyte.

Use of hydrophilic nanoparticles provides higher surface roughness on the substrate. Studies have demonstrated that the use of Fe-nanoparticles fabricated by evaporation and annealing of a 4 nm Fe layer at 600 °C affects the adhesion and uniformity of the spin-coated electrodes [10]. Figure 4a shows the atomic force microscopy (AFM) profiles of the Fe and bare SiO_2_ surfaces on 1 cm^2^ chips and the optical micrograph of the subsequent spin-coated wafer. The Fe surface demonstrates an average roughness of 2.1 nm, while the SiO_2_ surface has a roughness of only 0.28 nm. The surface on the roughened wafer is completely covered with a pink hue, while the non-roughened wafer is covered only in some areas. Figure 4b shows the percentage coverage of the spin-coated electrodes on different surfaces (percentages calculated based on optical microscopy of the surfaces). The surface with nanoparticles demonstrates the highest coverage, whereas the SiO_2_ (control) substrate has the lowest coverage on the entire wafer. The experiments performed include the spin-coating on an un-roughened surface (control group in red), a roughened surface without current collectors (blue), a roughened surface with Ti/Au current collectors (yellow), and 3 roughened surfaces with Ar plasma bombardment as a post-treatment to attempt to further enhance roughening. Ar post-treatments were attempted at durations of 3, 6, and 10 minutes (teal, grey-blue, and purple, respectively). The experiments pertaining to coverage demonstrate that the use of Ar ions to further roughen the surface proves counter intuitive, as they reduce the surface roughness and, thereby, the percentage coverage of the spin-coated electrode material on the entire wafer. Furthermore, increased roughness leads to a larger capacitance of the MSCs. Figure 4c shows the cyclic voltammograms of the roughened and non-roughened (Si/SiO_2_/Ti-Au surfaces) MSCs and clearly indicates a larger charge storage for the roughened devices. The capacitance for the MSCs was calculated from the cyclic voltammetry measurements using $C=\frac{Q}{\left(2*\Delta V\right)}$ where *Q* is the total charge accumulated over a ΔV voltage potential. The factor of 2 in the denominator comes from the average of the charging and the discharging area of the current curves. The areal capacitance ($C_{A}$) of an individual device was calculated by $C_{A}=C/A$, where A is the area of the MSC. Most of the devices fabricated on the roughened surfaces demonstrate a 1.5x improvement in the areal capacitance across all scan rates, from 0.1 V s^−1^ to 5 V s^−1^. A large capacitance directly translates into a higher energy and power densities for the roughened MSCs, as shown in Figure 4d. The energy density is calculated from $E=\frac{1}{2}C_{A}\Delta V^{2}$ and the power density is acquired from $P=\frac{E}{t}$, where t is the discharge time. Thus, enhancing the surface roughness through nanoparticle deposition can improve the performance of MSCs (regardless of device topology), make the fabrication steps more stable, and lead to a greater device yield with a uniform controllable performance.

### 3.2. Electrode Doping for Performance Enhancement

**Figure 5:**
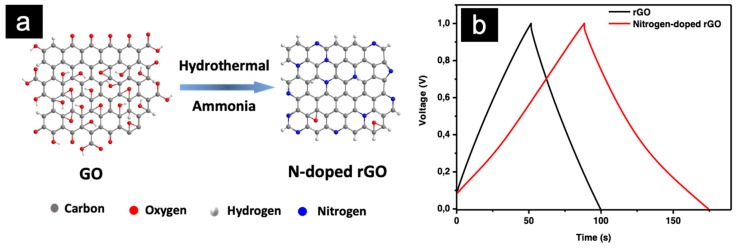
(**a**) Preparation of nitrogen-doped rGO from graphene oxide (GO) and ammonia solution (as the nitrogen precursor) through a hydrothermal process; (**b**) a galvanostatic charge–discharge (GCD) plot of rGO and a nitrogen-doped rGO.

Chemical doping of carbon-based nanomaterials with heteroatoms (B, N, O, S, P) has attracted tremendous attention due to their improved physicochemical properties and better energy density performance compared with undoped samples [68]. Among them, nitrogen-doped, carbon-based nanomaterials have shown superior electrochemical performances as electrode materials in supercapacitors. In general, nitrogen doping leads to the formation of different nitrogen sites, such as pyrrolic N, pyridinic N, and quaternary N/graphitic N [69]. This improves the active surface area and thereby the wettability by introducing extrinsic defects in the carbon networks; contributes to the surface faradaic reactions; and finally leads to an enhanced electrochemical performance. Chemical treatment of graphene, for example, GO with reactive nitrogen sources such as urea, melamine, polyaniline, or ammonia is an effective way to obtain nitrogen-doped rGO. As shown in Figure 5a, we have prepared nitrogen-doped rGO through a hydrothermal process, by reacting GO with ammonia solution at 180 °C, overnight. The content of nitrogen in the N-doped rGO could be up to 5.8%. As a control, GO was also reduced to rGO using the same reaction condition, without the addition of ammonia solution. Then, both rGO and nitrogen-doped rGO were employed as electrode materials in a two-electrode configuration for supercapacitor measurements. Figure 5b shows the galvanostatic charge–discharge (GCD) plot of rGO and nitrogen-doped rGO, at a current density of 0.5 A g^−1^. The specific capacitance was calculated to be 98, 176 F g^−1^ for rGO, and N-doped rGO, respectively. Compared with rGO, N-doped rGO showed an improved electrochemical performance due to the introduction of nitrogen doping.

### 3.3. Geometric Scaling of Electrodes

**Figure 6:**
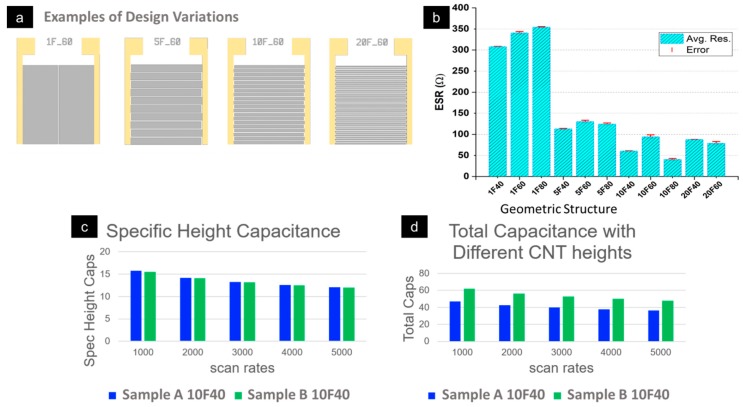
(**a**) 2D structural variations of the collectors and carbon-based electrodes of the MSCs where 1F, 5F, etc. denotes the number of fingered pairs of the collectors and electrodes and ‘60’ represents the spacing (in µm) between the fingers. (**b**) Measured average equivalent series resistance (ESR) for different collectors and electrode geometries representing 19 devices and 57 measurements. (**c**) Specific capacitance normalized for electrode height for two samples of different heights (3–4 μm for sample A and 5–6 μm for sample B). (**d**) Total capacitance for the two samples of different electrode heights (3–4 μm for sample A and 5–6 μm in B).

The capacitance of an MSC is critically dependent on the total electrode surface area. Consequently, the pore structure of the respective carbon-based material is important, as well as the geometric structure of the collectors and the electrodes. In order to accurately engineer a device with the desired capacitive behavior, we must also understand this scaling. Therefore, we have undertaken an extensive exploration of device scaling principles for both the areal geometry of the collectors and the electrodes, as well as the electrode height differences. This provides a good foundation for extrapolating how performance would be improved through modification of the collector and electrode geometry. Figure 6a displays the geometries investigated.

Figure 6b represents the measured average equivalent series resistance (ESR) for different geometric structures—suggesting that the collector and electrode geometry affects the power density. A total of 57 measurements were performed on 19 devices. Figure 6c and d are capacitance comparisons for the two samples with different electrode heights. Sample B is a thicker deposited electrode layer than Sample A (3–4 μm for sample A and 5–6 μm for sample B) and, thus, has a higher total capacitance. Significantly, when normalized for the height (Figure 6c), the specific capacitance is relatively constant—meaning that there is good penetration of the ions into the electrode and that the height scaled very well with the predicted trends (Figure 6d). In summary, we find that both energy and power density are affected by device geometry. This scalability of device properties is important for the design of future integrated MSC systems, which must follow spatial design constraints while fulfilling performance specifications. Without investigation into scaling, such device engineering would be unreliable. Here, we present the first steps toward understanding those scaling principles.

### 3.4. Separator Considerations for Stacked MSC Configurations

The electrode materials and electrolytes are the most influential constituents of a high performance MSC device. As a consequence, significant research has been focused on improving the capacitive performance by exploring different kind of electrode and electrolyte materials and designs [31,44,70]. Further, for planar MSCs, the investigation of separators is unnecessary (as the separator is special), while for stacked MSC configurations the separator is more important. Although planar MSCs are the primary focus of this article, some discussion of separators and ways to improve them is merited for providing a more complete overview of performance improving device metrics for both planar and stacked configurations.

A separator, an electrically insulating but ionically conducting membrane, is placed between two electrode materials of a device, in order to prevent electrode short circuit, while allowing an electrolyte ion diffusion with minimal obstruction. Therefore, the separator holds a critical role to suppress the failure of the MSC and to ensure a safe operation, which is especially important for applications such as medical devices. In addition, the separator could also make a significant difference for the MSC device performance by means of certain characteristic properties, such as thickness, porosity, wettability, chemical, and thermal stability. Ideally, a separator should hold certain properties as listed below [71]:

- **Thickness:** It should be as thin as possible without compromising mechanical stability or electrical reliability.
- **Chemical stability:** It should not react with the electrodes or the electrolyte under any circumstances. Otherwise, separator membrane degradation will compromise the reliability of the MSC device.
- **Porosity:** Porosity determines the amount of electrolyte the separator can hold in its pores to facilitate better ionic conductivity. At the same time, a highly porous separator might degrade under the influence of electrolytes. The standard porosity of typical separators is about 40%.
- **Pore Size:** Pore size should be in accordance with the pore size of the electrodes. For better performance, the ion size of the electrolytes should also be taken into account. In practice, separator membranes with sub-micron pore size are sufficient.
- **Wettability:** The separator should be easily wetted by aqueous electrolyte (hydrophilic) or non-aqueous electrolyte (hydrophobic) and should reliably hold the electrolyte for a very long time.
- **Permeability:** The presence of the separator should not influence the characteristics of the electrolyte. The loss of ionic conductivity can be expressed with a parameter called the Macmulin number. This is calculated as the ratio between the resistance of electrolyte in presence of a separator and the resistance of a pristine electrolyte. The Macmulin number of practical separators is of the order 10–12. Homogeneous composition of the separator material is also important to achieve an improved permeability.

We carried out a comparative electrochemical study of supercapacitors containing activated carbon (AC) electrodes, 1-ethyl-3-methylimidazolium acetate (EmImAc) ionic liquid electrolyte, and three different separators—(1) an electrospun polyvinylpyrrolidone (PVP) membrane with a thickness of 90 µm; (2) a commercially available glass fiber (GF) with a thickness of 200 µm; and (3) a Celgard membrane with a thickness of 25 µm. The AC electrode materials were synthesized according to the procedure mentioned in [72]. The supercapacitor test device was prepared with a two-electrode Swagelok cell containing a SS316 current collector. Three different electrochemical measurements were conducted—(1) GCD, (2) electrochemical impedance spectroscopy (EIS), and (3) open circuit voltage (OCV) decay. All electrochemical measurements were conducted at room temperature with a Gamry Reference 3000AE potentiostat/galvanostat. 

**Figure 7:**
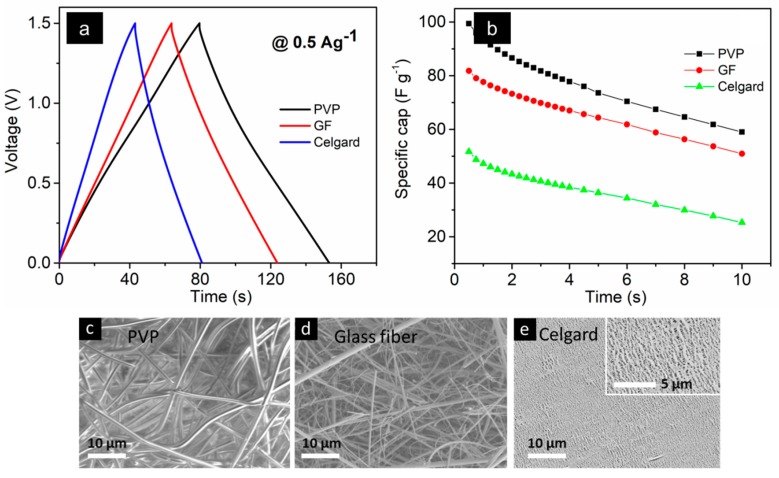
Measurements of supercapacitor devices including (**a**) GCD plots and (**b**) capacitance variations at different current densities. The measurements were done on an MSC with an AC electrode, a 1-ethyl-3-methylimidazolium acetate (EmImAc) ionic liquid electrolyte, and three different separators. (**c**–**e**) SEM images of polyvinylpyrrolidone (PVP), Glass fiber, and Celgard separators, respectively.

Figure 7a illustrates the GCD plots of the supercapacitors at a current density of 0.5 A g^−1^. All curves followed a triangular shape representative of a typical capacitive behavior. Longer charging and discharging times for the PVP-containing supercapacitor demonstrated a superior performance as compared to the commercial separators. As a consequence, the specific capacitance at 0.5 A g^−1^ was calculated to be 99 F g^−1^ for the PVP-containing MSC while it was 82 and 52 F g^−1^ for the MSCs-containing GF and Celgard, respectively. The specific capacitance was calculated according to the equations mentioned in [72]. Figure 7b shows the variation of the specific capacitance over time for the three different separators. Accordingly, a rate capability (capacitance at 10 A g^−1^ relative to 0.5 A g^−1^) was calculated to be 60% for the PVP, compared to 62% and 49% for the GF and Celgard-containing MSCs, respectively. Figure 7c–e display the SEM images of the PVP, Glass fiber, and Celgard separators, respectively. 

**Figure 8:**
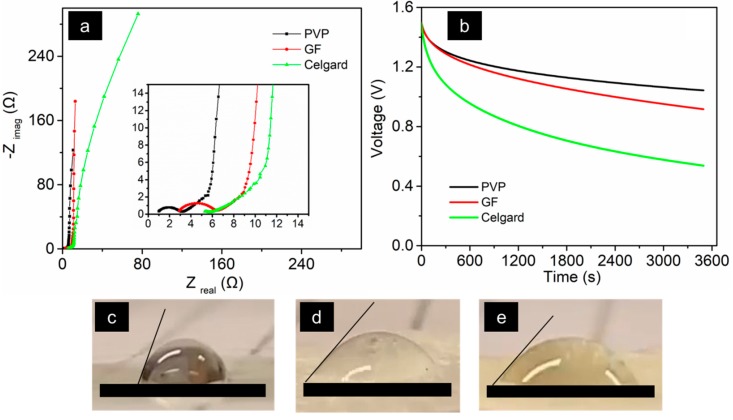
(**a**) Nyquist plot of the MSCs (same devices as in Figure 7). (**b**) Open circuit voltage (OCV) decay over time. The measurements used an AC electrode, 1-ethyl-3-methylimidazolium acetate (EmImAc) ionic liquid electrolyte, and three different separators. (**c**–**e**) The optical images used for the preliminary contact angle measurements of PVP, Glass fiber, and the Celgard separators, respectively.

Figure 8 shows an electrochemical impedance spectroscopy graph (Nyquist plot), which was used to extract the equivalent series resistance (ESR) values of the devices (first intersection of the semi-circle in the *X*-axis represents the ESR). The ESR of the PVP-containing supercapacitor is smaller (0.7 Ω cm^2^), as compared to the GF- and Celgard-containing supercapacitors, 2.2 and 3.6 Ω cm^2^, respectively. This was due to the good wettability and excellent porosity of the electrospun PVP separator membrane. The wettability of the separators were observed during electrolyte injection to be in the decreasing order for PVP, GF, and Celgard, respectively. Figure 8b shows the OCV decay (self-discharge) behavior of the MSCs. The voltage retention at the end of a one-hour interval was calculated to be 70% for the PVP-containing supercapacitor and 61% and 36% for the supercapacitors-containing GF and Celgard, respectively. In order to observe the wettability, a tentative contact angle measurement was carried out by injecting the EMIM Ac electrolyte in all three different separators. Figure 8c–e show the contact angle of the Celgard, Glass fiber, and PVP separators, respectively. For Celgard, the contact angle was measured to be 68° after 30 seconds of pouring the electrolyte droplet. The contact angles for Glass fiber and PVP separators were 46° and 45°, respectively, after just 3 seconds of pouring the electrolyte droplet and most of the electrolytes were absorbed by the end of 10 secs. Therefore, both the Glass fiber and PVP separators have superior wettability than the Celgard separator.

Based on the above measurement results, electrospun PVP membranes could be considered as a promising separator material for MSCs. This is significant as electrospinning is a potentially attractive deposition method for the realization of MSCs based on stacked devices configurations. However, further material characterizations are required to fully understand the improved capacitive performance, compared to the commercial separators—as well as the development of more comprehensive device manufacturing schemes.

### 3.5. Electrode/Current Collector Contact Resistance

Contact resistance is a critically important feature in any microscale device [73,74]. For MSCs, the contact resistance between the electrode and current collector is important because it will contribute to energy losses during charging and discharging. This will affect the actual energy density of the device as well as the power density. In order to characterize and engineer a good electrical contact between carbon-based electrode materials and the current collector, we have fabricated transmission line model (TLM) characterization devices to evaluate the contact resistance between gold contacts and chemical vapor deposited (CVD) graphene, VG and rGO, respectively. 

**Figure 9:**
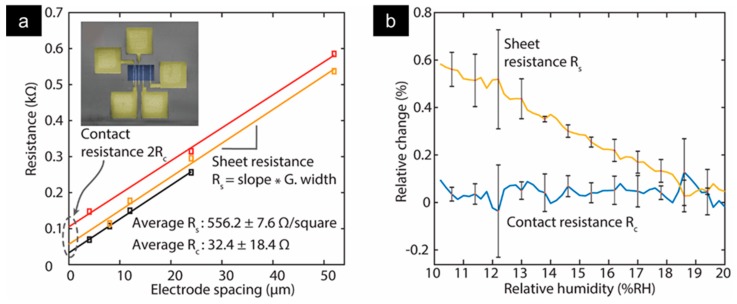
(**a**) Extraction of contact resistance of three transmission line model (TLM) devices with chemical vapor deposited (CVD) graphene patches on gold contacts. Extrapolating the linear fit (solid lines) of the measured resistance between the neighboring electrodes yields the contact resistance at the graphene/metal interface. Multiplication of the slope of the fit by the graphene patch results in the graphene sheet resistance. The inset displays a colorized scanning electron microscopy (SEM) photograph of a TLM device with gold contacts (yellow) and covering a graphene patch (blue). (**b**) Relative change in contact resistance and sheet resistance for variation in relative humidity (RH). Solid lines are the average of the three devices with 1σ error bars.

A TLM device consists of multiple gold electrodes of various spacing that are placed on an insulating substrate (inset of Figure 9a). A patch of graphene covers the electrodes and forms an electrical connection between the electrodes. The electrical resistance between two neighboring electrodes is composed of the resistance of the intermediate graphene patch and the contact resistance between graphene and gold in both contact areas. To extract the contact resistance, the measured resistance between two neighboring electrodes is plotted against the respective electrode spacing. The contact resistance is derived by extrapolating the residual resistance at zero contact-spacing. In addition, multiplication of the width of the graphene patch with the slope of the linear fit yields the sheet resistance of the intermediate graphene patch. As an example, Figure 9a shows the extraction of contact resistance ($R_{c}$) and sheet resistance ($R_{s}$) of CVD graphene patches on gold contacts for three different TLM devices. Here, $R_{c}$ averages to 32.4 ±18.4 Ω per contact and $R_{s}$ to 32.4 ± 18.4 Ω per contact and *R_s_* to 556.2 ± 7.6 Ω/*square*. 

Another important feature is the influence of moisture as well as the presence of the electrolytes on the contact resistance of the device. Moisture has been shown to have a strong influence on electrolyte behavior [75] but there are no studies, to our knowledge, that specifically address the influence of moisture on the contact resistance in MSC devices. Only one study examined the influence of moisture on contact resistance in graphene devices [76]. As such, this work continues the progress already made in examining contact resistances between the electrode material and the gold current collectors, and provides a preliminary investigation of the influence of low levels of moisture on the contact resistance. 

The same TLM devices with CVD graphene patches as in the inset of Figure 9awere measured at various humidity levels. The relative changes in *R_c_* and *R_s_* were plotted against the relative humidity (RH) (Figure 9b). The sheet resistance showed a significant increase of 0.6% when decreasing the RH from 20*%RH* to 10*%RH* whereas the contact resistance remained unaffected at these moisture levels. Note, both figures were extracted simultaneously from the very same measurement cycle. There is much room for future investigation (higher moisture concentration, in situ measurements of contact resistance in various electrolytes). However, this work aimed to provide some initial indication of graphene’s insensitivity to moisture and establish a foundation for future investigations to build upon.

## 4. Discussion

**Figure 10:**
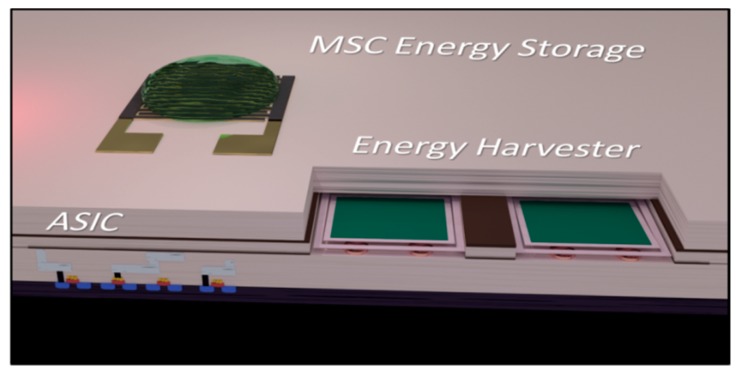
Schematic of a potential future self-powering system consisting back end of the line (BEOL) integration of an microsupercapacitors (MSC) and harvester with application specific integrated circuit (ASIC) components.

As previously mentioned, the primary application for MSCs is in on-chip energy storage components integrated alongside micro-energy harvesters [17,18,19,20]. For the overall device integration, the MSC would likely be built on top of a traditional CMOS stack containing digital logic, including power management circuitry and rectifiers. Energy harvesters and sensors could be fabricated in a back end of the line (BEOL) process and connected to power the CMOS stack through metal vias. In one possible integration scheme, MSCs could be integrated on-chip with other application specific integrated circuit (ASIC) components using a wafer bonding process—forming a self-powering microsystem (schematic of potential future integration scheme displayed in Figure 10). Such a design has, to our knowledge, not been integrated into a single on-chip microsystem.

## 5. Conclusions

We presented a number of design investigations and improvements for the realization of high-performance MSCs that can be integrated with CMOS-based integrated circuits. The proposed MSCs feature carbon-based electrode materials and can be integrated at the back end of the line of the integrated circuit fabrication to realize microsystems for future self-powering devices. Several critical design features are highlighted that influence both performance and integration potential. These include careful design of current collector and electrode interfaces to improve energy density, minimize contact resistance, and improve adhesion and uniformity. We further propose a CMOS-compatible manufacturing and integration scheme for realizing a fully on-chip, self-powering microsystem that can benefit from the aforementioned improvements.
